# Research on the development and innovation of online education based on digital knowledge sharing community

**DOI:** 10.1186/s40359-023-01337-6

**Published:** 2023-09-28

**Authors:** Xi Huang, Hongwei Li, Lirong Huang, Tao Jiang

**Affiliations:** 1 School of Economics and Management, Xiamen University of Technology, Xiamen, 361000 China; 2 Zhejiang Industry &Trade Vocational College, Wenzhou, 325000 China; 3 School of Marxism, Xiamen University of Technology, Xiamen, 361000 China; 4 School of Marxism, Huaqiao University, Xiamen, 361000 China

**Keywords:** Digital knowledge-sharing communities, Online education, Collaboration, Innovation, Student success, Thematic analysis, Qualitative research

## Abstract

**Background:**

Digital knowledge sharing (DKS) communities have emerged as a promising approach to support learning and innovation in online higher education. These communities facilitate the exchange of knowledge, resources, and ideas among educators, students, and experts, creating opportunities for collaboration, innovation, and lifelong learning. However, the impact and role of DKS communities in online education are not well understood, and further research is needed to explore their potential benefits and challenges.

**Purpose:**

This multi-objective qualitative study aims to investigate the impact and role of DKS communities in online higher education, identifying the factors that promote student success and the implications for the development of online education. The study collected data from 20 informants who have experienced teaching online during and after the pandemic. Data were collected through in-depth interviews and analyzed using thematic analysis. The informants were selected through theoretical sampling.

**Methodology:**

To explore the impact and role of DKS communities in online higher education, this study employed a multi-objective qualitative research method. Data were collected through in-depth interviews conducted with 20 informants who possessed experience in teaching online during and after the pandemic. The informants were selected through theoretical sampling to ensure diverse perspectives and insights. The collected data were subsequently analyzed using thematic analysis, allowing for the identification of key themes and patterns.

**Findings:**

The findings of this study provide valuable insights into the impact and role of DKS communities in online higher education. These insights encompass various aspects, including the benefits and challenges of DKS in online education, the factors that contribute to student success, and the implications for the ongoing development and innovation of online education.

**Conclusions:**

In conclusion, this multi-objective qualitative study sheds light on the significance of DKS communities in online higher education. It underscores their potential to enhance collaboration, innovation, and lifelong learning. The findings also emphasize the importance of addressing challenges and fostering an inclusive and supportive online learning environment. These insights inform best practices and contribute to the continuous development and innovation of online education, particularly in the post-pandemic educational landscape.

**Supplementary Information:**

The online version contains supplementary material available at 10.1186/s40359-023-01337-6.

## Introduction

Education for sustainable development is a vital aspect of achieving the Sustainable Development Goals proposed by the United Nations Economic and Social Development Organization (UNESCO) and adopted by institutions worldwide [1]. Education is seen as an essential means of creating awareness and promoting sustainable development by encouraging individuals to adopt environmentally friendly behaviors. The concept of Higher Education for Sustainable Development (HESD) has been widely discussed in recent years [2]. Higher education institutions, such as universities and technical training colleges, have gradually become essential platforms for promoting sustainable development in the 21st century [3].

Digitalization, on the other hand, has silently revolutionized the way humans live. Almost all fields of knowledge are benefiting from digitalization, including education, healthcare, business, and entertainment [4]. Digitalization has provided a more efficient way of knowledge sharing, allowing individuals to access and share information quickly and easily. This has enabled education to be more widespread and accessible to individuals worldwide, creating opportunities for people to improve their knowledge and skills [5]. Institutions of higher education have been at the forefront of digitalization, transforming the way instructors develop courses and disseminate research findings. Digital networks such as 5G are gradually rolling out worldwide, enabling faster and more reliable communication, which has transformed many industries, including the industrial sector. Universities and technical training colleges have played a significant role in advancing UNESCO’s Sustainable Development Goals, with many initiatives launched worldwide to promote its further development [1].

According to Elmassah et al. [4] higher education has traditionally been the primary platform for generating, developing, and promoting knowledge. In recent years, countries such as China, India, Thailand, Vietnam, Nigeria, and Kenya have successfully applied digitalization and used higher education to promote sustainable development [6, 7]. Digitalization has been a powerful tool for sharing knowledge, as noted by Gregson et al. [8]. With the advent of the internet and higher technology, institutions of higher education can share knowledge generated by experts with new generations of learners [9]. As Funk [10] suggests, “sharing knowledge is power,” and digitalization provides an effective means of achieving this.

Digital technology is transforming how learners understand and interpret new knowledge, as well as impacting the motivation of academics to share their research findings. The construction industry [11, 12] and the information technology field [12] are examples of how digitalization is changing the way people work. This is due to the increased flow of information made possible by digitalization, which enables better coordination between independent units and individuals. In higher education institutions, digitization has had a significant impact, with several initiatives launched worldwide to promote its further development [13]. Digital platforms provide new structures for better knowledge sharing and continuous innovation, as noted by Arfi et al. [14].

Technology can be used to create Digital Knowledge Sharing (DKS) communities (DKSCs). These communities enable collaboration and networking among learners and educators, facilitate personalized learning, incorporate gamification to enhance the learning experience, leverage artificial intelligence and machine learning to provide adaptive learning experiences, and require quality control to ensure the accuracy and reliability of learning resources [15–21]. Digital knowledge sharing in online education has several benefits, including accessibility, flexibility, cost-effectiveness, customization, and interactive learning [22, 23]. This approach has the potential to transform traditional education by providing students with a flexible, accessible, and engaging learning experience.

Digitalization is portrayed as an omnipresent force that has revolutionized various sectors, including education [15]. The literature underscores the advantages of digital technology in facilitating knowledge sharing, making education more accessible, and fostering global connectivity. Nonetheless, it is crucial to acknowledge the digital divide that persists globally, wherein not all individuals have equitable access to digital resources and technologies. Therefore, the assertion that digitalization universally enhances accessibility should be tempered with an awareness of existing disparities [16, 17].

Grounded in the belief that collaborative learning, dissemination of best practices, and technological innovation are central components of educational progress, this research seeks to explore how DKS communities act as catalysts for continual improvement in the digital education landscape. Drawing from theories of educational technology, social learning, and innovation diffusion, the study aims to elucidate the multifaceted ways in which these communities enhance the online learning experience, foster critical thinking, and promote a culture of lifelong education [17]. By investigating the interplay between technology, collaborative learning, and innovation within the context of online education, this study endeavors to contribute to the theoretical foundations underpinning the advancement of digital learning environments [17].

Furthermore, the literature review highlights the transformative potential of digital technology in higher education, particularly in terms of knowledge dissemination and collaboration among learners and educators. It suggests that digital platforms can facilitate continuous innovation and personalized learning experiences [12–18]. However, the review does not thoroughly address the challenges and drawbacks of this digital transformation. Issues related to digital literacy, data privacy, and the quality of online education resources require careful consideration. Additionally, the potential homogenization of education through digitalization, where diverse perspectives may be marginalized, warrants scrutiny.

The concept of DKS communities is introduced as a means to harness the power of digital technology in education. These communities are presented as innovative solutions for collaboration, gamification, and adaptive learning. While the potential benefits of DKSCs are intriguing, the review does not offer a comprehensive examination of the effectiveness of such communities in practice. Are DKS communities accessible to all students, and do they effectively enhance learning outcomes? These questions remain unanswered.

In conclusion, the literature review provides a compelling narrative about the transformative potential of digitalization in higher education for sustainable development. However, it is important to approach this paradigm shift with critical scrutiny. Equitable access, digital literacy, quality assurance, and the preservation of diverse educational experiences are paramount considerations in the era of digital education. The promising fusion of education and digitalization should be tempered with a commitment to addressing the challenges that arise in this evolving landscape.

As we stand at the intersection of technology and education, it becomes increasingly evident that our ability to harness these innovations will have far-reaching implications for the future of learning. In this context, understanding the intricate relationship between cognitive control, relational aggression, and emerging learning technologies among sportsmen adults is not just academically valuable but also relevant to the broader discourse on how technology is shaping the educational landscape. By delving into this subject matter, we aim to contribute to the ongoing dialogue on educational innovation, shedding light on the ways in which emerging technologies impact cognitive processes and social dynamics within the context of sports education. Specifically, there is a need to understand the challenges and opportunities of using these communities to develop and innovate online education. This study aims to contribute to the growing body of research on the development and innovation of online education based on digital knowledge sharing. This research can inform educators and policymakers on how to best leverage DKS communities to enhance the quality and effectiveness of online education in the post-pandemic era. Therefore, this research aims to investigate the following research questions:

1. What are the benefits and challenges of digital knowledge sharing in online education?
2. What is the role of DKS communities in the development and innovation of online education?
3. How do DKS communities impact student learning and engagement in online education?
4. What factors contribute to the effectiveness of DKS communities in promoting student success?

## Research method

### Research design

The research design for this study was a qualitative research method using a phenomenological approach. “Phenomenology is particularly useful in research studies that aim to explore subjective experiences and perceptions of participants. It allows the researcher to gain a deep understanding of the lived experiences of the participants, and to uncover the meaning and essence of those experiences [24]. The study aimed to explore the experiences of university professors in higher education in China regarding the role and contribution of DKS communities to online education. The phenomenological approach allowed the researcher to understand the subjective experiences and perceptions of the informants regarding the use of DKS communities in online education.

### Informants

The informants for this study were 20 university professors of higher education in China who were selected through theoretical sampling. The selection criteria for the informants were that they have experience in teaching online courses and have used DKS communities in their teaching. The rationale for sample size was data saturation which occurred when the 20^th^ informant was interviewed. The informants were selected from Tsinghua University, Peking University, Fudan University, Nanjing University, and Wuhan University. The average age of the group was 30 years old with a standard deviation of 5 years, while the average number of years of teaching experience was 12 with a standard deviation of 5 years. The informants who participated in the study did so willingly and were aware of the purpose of the research. They were provided with information about the study and provided their informed consent before being interviewed. The confidentiality and privacy of the informants and the data collected were ensured throughout the research process. The informants were assured that their personal information and responses would be kept confidential and that their identities would not be revealed in any publications or reports resulting from the study. All data collected during the study was stored securely and only accessed by the research team. These measures were taken to ensure that the informants felt comfortable sharing their experiences and perceptions and to protect their privacy and confidentiality.

### Instrumentation

The primary instrument for data collection in this study was semi-structured and focused interviews. The semi-structured interviews allowed the researcher to explore the informants’ experiences and perceptions regarding the use of digital knowledge-sharing communities in online education in a flexible and open-ended manner. The focus interviews allowed the researcher to probe more deeply into specific topics or issues related to the use of digital knowledge-sharing communities in online education (See Additional file 1: Appendix A).

### Data collection procedure

The informants who participated in the study were recruited through theoretical sampling, which involved identifying potential participants who met the selection criteria and inviting them to participate in the study. The selection criteria included having experience in teaching online courses and using digital knowledge-sharing communities in their teaching. The informants were selected from several universities, including Tsinghua, Peking, Fudan, Nanjing, and Wuhan. Once the informants were recruited, they were given information about the study and provided their informed consent before being interviewed. The interviews were conducted either face-to-face or online, depending on the availability and preference of the informants. For the face-to-face interviews, the researcher arranged to meet with the informants at their universities or other convenient locations. For the online interviews, the researcher used video conferencing applications such as Skype or Zoom.

The interviews were semi-structured and focused on the research questions, with the aim of exploring the experiences and perceptions of the informants regarding the role and contribution of digital knowledge-sharing communities to online education. The interviews were audio-recorded with the consent of the informants and later transcribed verbatim for analysis. In addition to the audio recordings, the researcher also took field notes during the interviews to capture non-verbal cues and contextual information.

Throughout the data collection process, the privacy and confidentiality of the informants and the data collected were ensured. The informants were assured that their personal information and responses would be kept confidential and that their identities would not be revealed in any publications or reports resulting from the study. All data collected during the study was stored securely and only accessed by the research team. These measures were taken to protect the privacy and confidentiality of the informants and to ensure that they felt comfortable sharing their experiences and perceptions.

### Data analysis

The data were analyzed using MAXQDA software (version 2022), following the recommendation of Creswell [24]. The unit of analysis in this study was the sentence, and the researcher focused on manifest content rather than latent content. The qualitative data were collected, analyzed, and reported in English. An inductive approach to content analysis was employed, as no preexisting theory or framework guided the generation of codes, categories, and themes [25]. Sutton and Austin [26] proposed a five-step process for qualitative data analysis, which was followed in this study. Firstly, the data were cleaned by addressing linguistic errors, ambiguities, inaccuracies, and repetitions. Secondly, the researcher read the data multiple times and developed open codes. Thirdly, the open codes were categorized as relevant axial codes or subtopics. Fourthly, the axial codes and subtopics were grouped under higher-order selective codes and general themes. Finally, a detailed report was prepared to document the completed data analysis process and its interpretation. The frequency of generated codes, topics, and categories was reported, and the results were visually presented using the MAXMAP properties of MAXQDA, creating visual representations. To ensure the credibility of the analytical process, 20% of the generated codes were randomly selected and re-coded by a second coder with sufficient knowledge and experience in qualitative research. Specifically, 80 codes were created, and 20 of them were sent to the second coder. Upon coding, the second coder disagreed with the first coder on one code, resulting in an intercoder agreement coefficient of 96%. The two coders discussed and resolved any disagreements by making the necessary changes, ensuring the completion of the qualitative data analysis process.

### Research quality

In order to uphold the integrity of the research conducted in this study, the researcher implemented various methodologies, such as member checking, peer debriefing, and reflexivity. Member checking is a method used to validate and authenticate data by consulting with the individuals who provided the information. This process aids in ensuring that the data accurately represents their experiences and perspectives. Peer debriefing is a process that entails soliciting feedback and input from fellow researchers or subject matter experts in order to enhance the credibility and reliability of the research findings. Reflexivity encompasses the critical examination of the researcher’s biases, assumptions, and values, and their potential impact on the research process and outcomes. This practice serves to enhance the study’s validity and reliability.

## Findings

The findings of the study are presented based on the order of the research questions.

### Research questions 1

The first research question aimed at exploring the benefits and challenges of digital knowledge sharing in online education. interviews with the informants were analyzed and 7 benefits and 10 main challenges were extracted. Below are explanations for each extracted theme using at least two citations, along with two quotations from informants for each theme:

A. Benefits of DKS communities

Detailed analysis of the interviews with the informants revealed that DKS communities have several benefits and challenges, each is explained and exemplified as follows.

1. Improved student engagement

Improved student engagement is a benefit of digital knowledge sharing in online education. DKS communities provide a platform for students to interact and collaborate with each other, which can improve their engagement and motivation to learn. According to one informant, “DKS communities help keep students engaged in the course material, which can lead to better learning outcomes” (Informant 1). Another informant stated, “When students are able to participate in online discussions and share their own ideas and perspectives, they become more invested in the learning process” (Informant 2).

2. Enhanced learning outcomes

Using DKS communities in online education can enhance learning outcomes by providing students with access to a wider range of resources and perspectives. According to one informant, “DKS communities can help students develop critical thinking skills by exposing them to diverse perspectives and encouraging them to challenge their own assumptions” (Informant 3). Another informant stated, “Using digital tools like online discussion forums and collaborative documents can help students engage more deeply with the material and apply what they’ve learned in new ways” (Informant 4).

3. Flexibility and accessibility

Flexibility and accessibility are benefits of digital knowledge sharing in online education. Online education allows for greater flexibility in terms of when and where learning takes place, which can be especially beneficial for students who have other commitments such as work or family responsibilities. According to one informant, “Online courses provide flexibility for students who might not be able to attend traditional in-person classes due to other commitments” (Informant 5). Another informant stated, “DKS communities make education more accessible to students who might not have access to traditional educational resources, which can help level the playing field for students from different backgrounds” (Informant 6).

4. Improved student outcomes

Digital knowledge sharing in online education can lead to improved student outcomes such as better grades, higher retention rates, and increased satisfaction with the learning experience. According to one informant, “Digital knowledge sharing can lead to better learning outcomes because students are able to access a wider range of resources and engage with different perspectives” (Informant 13). Another informant stated, “Online education can be especially beneficial for students who might struggle with traditional classroom settings, as it can provide a more personalized and flexible learning experience” (Informant 14).

5. Increased collaboration

DKS communities can facilitate increased collaboration among students and instructors. According to one informant, “Online discussion forums and collaborative documents can allow students to work together on projects and assignments, which can enhance their understanding of the material and improve their communication skills” (Informant 15). Another informant stated, “Digital knowledge sharing can create a sense of community among students who might not have had the opportunity to interact with each other otherwise” (Informant 16).

6. Personalization of learning:

Digital knowledge sharing in online education can enable a more personalized learning experience for students. According to one informant, “Online courses can allow students to work at their own pace and focus on the areas where they need the most help, which can lead to better learning outcomes” (Informant 11). Another informant stated, “Digital knowledge sharing can allow instructors to provide more targeted feedback to individual students, which can help them improve their understanding of the material” (Informant 18).

7. Greater feedback and assessment opportunities:

DKS communities can provide greater opportunities for feedback and assessment. According to one informant, “Online quizzes and assessments can provide immediate feedback to students, which can help them identify areas where they need to improve” (Informant 19). Another informant stated, “Digital knowledge sharing can allow instructors to provide more frequent and detailed feedback to students, which can help them stay on track and improve their performance” (Informant 20). Despite the benefits of the DKS communities, the informants mentioned some challenges, which are exemplified and explained as follows.

B. Challenges of DKS communities

Informant have stated some challenges of DKS communities, which are explained and exemplified as follows.

1. Technical difficulties

Technical difficulties are a challenge of digital knowledge sharing in online education. Students and instructors may encounter technical difficulties such as internet connectivity issues or software glitches, which can disrupt the learning process and create frustration for students and instructors alike. According to one informant, “Technical difficulties can be a major barrier to effective online learning, and can lead to students feeling frustrated and disengaged from the course material” (Informant 7). Another informant stated, “Instructors need to be prepared to troubleshoot technical issues and provide support to students who are experiencing difficulties with the digital tools” (Informant 8).

2. Maintaining academic integrity

Maintaining academic integrity is a challenge of digital knowledge sharing in online education. DKS communities can create challenges around maintaining academic integrity, as students may be tempted to plagiarize or share answers with each other. According to one informant, “Ensuring academic integrity in DKS communities requires a concerted effort from instructors and students to communicate expectations and uphold ethical standards” (Informant 9). Another informant stated, “Instructors need to be vigilant about monitoring student behavior in DKS communities to ensure that academic dishonesty is not taking place” (Informant 10).

3. Digital divide

The digital divide is a challenge of digital knowledge sharing in online education. Not all students have equal access to technology and internet connectivity, which can create a digital divide in online education. According to one informant, “The digital divide can exacerbate existing inequalities in education and limit opportunities for students who lack access to the necessary technology and resources” (Informant 11). Another informant stated, “Institutions need to be mindful of the digital divide and take steps to ensure that all students have access to the technology and resources necessary to participate in online learning” (Informant 12).

4. Social isolation and lack of interaction

DKS communities can create a sense of social isolation and limit opportunities for interaction among students and instructors. According to one informant, “Online education can be a lonely experience for students who are used to traditional classroom settings, as they may miss out on the social interactions that are a key part of the learning experience” (Informant 17). Another informant stated, “Instructors need to be intentional about creating opportunities for interaction and collaboration among students in online courses” (Informant 20).

5. Time management and self-discipline

Digital knowledge sharing in online education can require strong time management and self-discipline skills, which can be challenging for some students. According to one informant, “Online courses require a high degree of self-discipline and time management skills, as students are often responsible for setting their own schedules and managing their own learning” (Informant 10). Another informant stated, “Instructors can help students develop these skills by providing clear expectations and deadlines, and by encouraging them to set goals and prioritize their workload” (Informant 4).

6. Limited access to hands-on learning experiences:

Digital knowledge sharing in online education can limit opportunities for hands-on learning experiences, which can be a challenge for students in certain fields of study. According to one informant, “Online education may not be suitable for certain fields such as science and engineering, where hands-on learning experiences are an important part of the curriculum” (Informant 3). Another informant stated, “Instructors need to be creative in finding ways to provide hands-on learning experiences in online courses, such as through virtual simulations or online labs” (Informant 14).

7. Quality control

The challenge of DKS communities is ensuring the quality and accuracy of the content being shared. With so much information available, it can be difficult to sift through it all and ensure that learners are accessing high-quality and reliable resources.” (Informant 14). Similarly, informant 18 has stated that, “in the era of DKS Communities, the abundance of information poses a formidable challenge: the assurance of content quality and reliability. Amidst the vast digital landscape, the task of curating high-quality resources becomes imperative to safeguard the learning journey.” (Informant 11).

8. Intellectual property

The issue of intellectual property is a complex one in the context of digital knowledge-sharing communities. It is important to ensure that content is properly attributed and that copyright laws are respected, but this can be difficult to enforce in online environments. (Informant 16). In addition, informant 15 stated, “In the realm of DKS communities, the intricacies of intellectual property come to the forefront. Balancing the imperative of proper attribution and the adherence to copyright laws with the challenges of enforcement in the vast online domain presents a multifaceted dilemma.”

9. Cultural differences

Cultural differences can present a challenge for digital knowledge-sharing communities, particularly when it comes to language barriers. It is important to ensure that these communities are inclusive of all cultures and languages and that learners have access to resources that are culturally relevant to them. This finding is in line with quotations from informant 9 who stated, “cultural diversity emerges as a compelling challenge, often manifesting through the formidable barriers of language. The imperative lies in fostering inclusive platforms that transcend cultural boundaries, granting learners access to resources imbued with cultural relevance.” Informant 5 also stated, “the harmonious coexistence of diverse cultures within digital knowledge-sharing communities highlights the importance of dismantling language barriers. Ensuring inclusivity through culturally relevant resources stands as an essential endeavor to bridge the gap in global education.”

### Research question 2

The second research question addressed the role of DKS communities in the development and innovation of online education. Interviews with the informants were analyzed and 7 themes were extracted, which are explained and exemplified as follows.

1. Facilitation of collaboration and innovation

DKS communities can facilitate collaboration and knowledge exchange among students and instructors, which can lead to the development of innovative approaches to teaching and learning in online education. According to one informant, “DKS communities can help to create a culture of collaboration and experimentation, where ideas can be shared and refined in real-time” (Informant 1). Another informant stated, “Collaboration is a key component of online education, and DKS communities can provide a platform for students and instructors to work together on projects and assignments, which can lead to the development of innovative solutions” (Informant 2).

2. Encouragement of innovation

DKS communities can encourage innovation in online education by providing a space for experimentation and exploration of new teaching methods and technologies. According to one informant, “DKS communities can encourage instructors to experiment with new technologies and teaching methods, which can lead to the development of more engaging and effective online courses” (Informant 3). Another informant stated, “Innovation in online education can lead to improved learning outcomes and greater student engagement, and DKS communities can play a key role in supporting this innovation” (Informant 4).

3. Dissemination of best practices

DKS communities can serve as a platform for disseminating knowledge about best practices and successful approaches to online education. According to one informant, “Sharing knowledge and experiences through digital platforms can help to build a community of practice around online education, and can lead to the development of new insights and approaches” (Informant 5). Another informant stated, “DKS communities can provide a way for instructors to learn from each other and to stay up-to-date on the latest trends and developments in online education” (Informant 6).

4. Support for lifelong learning

DKS communities can play a role in supporting lifelong learning by providing access to a wide range of learning resources and opportunities. According to one informant, “DKS communities can provide a platform for individuals to continue learning throughout their lives and can help to bridge the gap between formal education and informal learning” (Informant 9).

5. Enhancement of critical thinking

DKS communities can enhance critical thinking skills by exposing students to diverse perspectives and encouraging them to engage in discussions and debates. According to one informant, “DKS communities can help to develop critical thinking skills by exposing students to a variety of viewpoints and challenging them to think deeply about complex issues” (Informant 10).

6. Promotion of student agency

DKS communities can promote student agency by giving them more control over their learning and encouraging them to take an active role in shaping their educational experiences. According to one informant, “DKS communities can give students a sense of ownership over their learning, and can help to foster a sense of autonomy and independence” (Informant 14).

7. Development of digital literacy

DKS communities can help to develop digital literacy skills by providing opportunities for students to engage with digital tools and platforms. According to one informant, “DKS communities can help to develop digital literacy skills by giving students the opportunity to interact with a variety of digital tools and platforms, and by encouraging them to experiment and explore” (Informant 18).

### Research question 3

The third research question aimed at exploring how DKS communities impact student learning and engagement in online education. Findings revealed that digital knowledge sharing (DKS) communities can have a significant impact on student learning and engagement in online education. Here are some potential ways that DKS communities can impact student learning and engagement:

1. Increased access to resources

DKS communities can provide students with access to a wide range of learning resources, including articles, videos, podcasts, and other multimedia. This can help to increase the diversity of perspectives and ideas that students are exposed to, enhancing the quality and effectiveness of their learning. For instance, informant 7 stated, “Being part of the DKS community has given me access to a wide range of resources that I wouldn’t have found on my own. It has helped me to deepen my understanding of the course material and to see things from different perspectives.”

2. Peer support and collaboration

DKS communities can provide opportunities for peer support and collaboration, which can help to promote student engagement and motivation. Students can ask questions, share insights, and work together on projects and assignments, fostering a sense of community and connection. This finding can be supported by a quotation from informant 2 who stated, “The DKS community has been a great way to meet new people and to work together on projects. It’s helped me to feel more connected to the course and to stay motivated.”

3. Diverse perspectives

DKS communities can expose students to diverse perspectives and ideas, which can help to broaden their understanding and deepen their critical thinking skills. Students can engage in discussions and debates with others who have different backgrounds and experiences, leading to a more well-rounded learning experience. The finding is supported by quotation from informant 7, who stated, “The DKS community has exposed me to a wide range of perspectives and ideas that I never would have encountered otherwise. It’s helped me to broaden my understanding of the course material and to think more critically about the issues.”

4. Active learning

DKS communities can promote active learning by providing opportunities for students to engage with course material in meaningful ways. Students can apply their learning to real-world scenarios, participate in simulations or case studies, and engage in hands-on activities that promote deeper understanding of course concepts. As an example, informant 5 stated, “The DKS community has been a great way to engage with the course material in a more meaningful way. It’s helped me to stay motivated and to feel like I’m making progress.”

5. Flexibility and personalization

DKS communities can provide flexibility and personalization in the online learning experience, giving students more control over their learning and allowing them to tailor their experience to their individual needs and preferences. This can help to increase engagement and motivation, as well as promote a sense of ownership and responsibility for learning. As an example, informant 9 stated, “The DKS community has been a great way to personalize my learning experience. I’ve been able to explore topics that interest me and to find resources that are relevant to my goals.”

### Research question 4

Research question 4 aimed at exploring the factors which contribute to the effectiveness of DKS communities in promoting student success. The theme analysis of the interviews with the informants showed 5 factors contribute to the effectiveness of DKS communities, each is explained as follows:

1. Active participation by students:

The data analysis revealed that active participation by students is a key factor that contributes to the effectiveness of DKS communities in promoting student success. Students who actively participate in these communities tend to have a better understanding of the course material and are more engaged in their learning. For instance, informant 8 argued, “Students who participate in the community are more likely to have a better understanding of the course material and are more engaged in their learning.”

2. Clear expectations and guidelines

The analysis also found that establishing clear expectations and guidelines for DKS community participation can help students understand what is expected of them and how they can contribute to the community. This can lead to greater engagement and participation by students, which in turn can contribute to their success. One of the informants stated. “I give clear guidelines and expectations to my students, and I encourage them to interact with each other and share their knowledge.”

3. Supportive and collaborative learning environment:

The findings suggest that a supportive and collaborative learning environment is another factor that contributes to the effectiveness of DKS communities in promoting student success. Students who feel supported and encouraged by their peers and instructors in these communities tend to be more motivated and engaged in their learning. For instance, informant 5 stated, “The DKS community is a place where students can learn from each other, share their experiences, and support each other.”

4. Relevance and usefulness of the DKS community

The analysis also revealed that the relevance and usefulness of the DKS community is important in promoting student success. Students are more likely to participate in these communities when they see the relevance and usefulness of the community in relation to their course goals and learning outcomes. To exemplify the theme, the following quotation is used, “The DKS community is a valuable resource for students to ask questions, share knowledge, and learn from each other.” (Informant 3).

5. Flexibility and adaptability of the DKS community:

Finally, the data analysis showed that the flexibility and adaptability of the DKS community is another important factor in promoting student success. DKS communities that are flexible and adaptable to the changing needs and preferences of students tend to be more effective in promoting student success. For instance, informant 19 stated, “I try to be flexible and adapt the community to the changing needs and preferences of my students.”

## Discussion

This multi-objective qualitative study at exploring the impact and role of DKS communities in innovation in online higher education. A qualitative research method was used and the interviews with 20 informants were analyzed. With regard to the first objective, the informants mentioned 7 benefits and 9 challenges of using DKS communities in the development and innovation of online education based on a digital knowledge-sharing community. The findings highlight the potential benefits and challenges of using DKS communities for developing and innovating online education, as well as the ways in which these communities can contribute to higher education sustainability. Recent research has supported these findings and provided further insights into the benefits and challenges of using DKS communities in education. For example, a study by Ansari, et al. [27] found that DKS communities can enhance collaborative learning and knowledge-sharing among students, leading to better learning outcomes. Similarly, a study by Cheng, et al., [28] found that DKS communities can improve teacher collaboration and professional development, leading to more effective teaching practices.

The findings related to the challenges are also consistent with the findings of some researchers. For example, a study by Nugroho, et al. [29] found that quality control remains a challenge for digital knowledge-sharing communities, with a need for better tools and strategies for evaluating the quality of shared resources. Additionally, a study by Zhao, et al., [30] found that the digital divide remains a major barrier to accessing digital knowledge-sharing communities, particularly for learners in rural areas or with limited access to technology. To address these challenges, recent research has proposed various solutions and strategies. For example, Similarly, a study by Thi Minh Ly, et al. [31] proposed a model for bridging the digital divide, which involves providing learners with access to digital infrastructure and training in digital literacy skills. Similarly, a study by Shawar et al. [32] proposed a framework for quality assurance in digital knowledge-sharing communities, which includes guidelines for content evaluation and quality control.

With regard to the second objective, the analysis of the interviews with informants in this study revealed seven themes related to the role of DKS communities in the development and innovation of online education. These themes include facilitation of collaboration and innovation, encouragement of innovation, dissemination of best practices, support for lifelong learning, enhancement of critical thinking, promotion of student agency, and development of digital literacy. The findings of this study are consistent with previous studies that have examined the role of DKS communities in the development and innovation of online education. For example, Dabbagh and Kitsantas [33] found that personal learning environments, which are similar to digital knowledge-sharing communities, can facilitate collaboration and knowledge exchange among learners and can enhance critical thinking and digital literacy skills. Similarly, Barab, et al. [34] found that online communities of practice can support professional development and knowledge sharing among educators.

The finding that DKS communities can encourage innovation is also consistent with previous studies. Siemens and Tittenberger [35] argued that emerging technologies, such as digital knowledge-sharing communities, can support innovation in education by providing a platform for experimentation and exploration of new teaching methods and technologies. Moreover, the finding that DKS communities can disseminate knowledge about best practices and successful approaches to online education is also consistent with previous studies. Palloff and Pratt [21] argued that online communities of practice can serve as a platform for sharing knowledge and experiences, leading to the development of new insights and approaches. Similarly, Garrison, et al. [36] found that computer conferencing can support knowledge sharing and dissemination among learners and educators. Furthermore, the finding that DKS communities can support lifelong learning is also consistent with previous studies. Warschauer and Matuchniak [37] argued that digital technologies can support lifelong learning by providing access to a wide range of learning resources and opportunities.

In addition, that DKS communities can enhance critical thinking skills is also consistent with previous studies. Hrastinski [38] argued that online learning can support critical thinking skills by providing opportunities for collaborative learning and discussion among learners. Similarly, the findings are consistent with Wang, et al., [39] found that social media, which are similar to digital knowledge-sharing communities, can promote student agency by giving students more control over their learning and encouraging them to take an active role in shaping their educational experiences. Finally, the finding that DKS communities can develop digital literacy skills is also aligned with previous studies. Siemans and Tittenberger [35] and Garrison, et al. [36] argued that emerging technologies, such as digital knowledge-sharing communities, can support the development of digital literacy skills by providing opportunities for learners to engage with digital tools and platforms.

The third objective was to delve into the impact of DKS on students learning in higher education. The findings of this study are consistent with previous studies that have examined the impact of DKS communities on student learning and engagement in online education. The first potential impact, increased access to resources, is supported by the findings of Palloff andPratt [21] and Garrison, et al. [36] who argued that digital technologies can support lifelong learning by providing access to a wide range of learning resources and opportunities. The second potential impact, peer support, and collaboration is supported by the findings of Warschauer, and Matuchniak [37] who found that online communities of practice can support professional development and knowledge sharing among educators. The third potential impact, diverse perspectives, is supported by the findings of Hrastinski [38] who argued that online learning can support critical thinking skills by providing opportunities for collaborative learning and discussion among learners. The fourth potential impact, active learning, is supported by the findings of Dabbagh and Kitsantas [33] who found that personal learning environments can enhance critical thinking and digital literacy skills. The fifth potential impact, flexibility, and personalization, is supported by the findings of Wang, et al., [39] who found that social media can promote student agency by giving students more control over their learning and encouraging them to take an active role in shaping their educational experiences.

With regard to the last question findings, it can be argued that the findings of this study are consistent with previous research on the importance of student participation in online learning communities. A study by Warschauer and Matuchniak [37] found that students who actively participated in online learning communities had higher levels of engagement and were more likely to succeed in their courses. Similarly, a study by Rovai and Jordan [40] found that students who were highly involved in online learning communities had higher levels of satisfaction and academic success.

The next finding was the importance of clear expectations and guidelines for online learning communities which has also been identified in previous research. For example, Richardson [41] found that providing clear guidelines and expectations for online learning communities can help students feel more comfortable and engaged in the learning process. Additionally, Palloff and Pratt [21] found that clear guidelines and expectations can help students understand how to participate in online learning communities and contribute to their success.

The significance of a supportive and collaborative learning environment has also been supported by previous research. A study by Garrison, et al., [36] found that students who felt supported and encouraged by their peers and instructors in online learning communities were more likely to be engaged and successful in their courses. Additionally, a study by Shea et al., [42] found that a sense of community and support was a key factor in promoting student success in online learning environments. Moreover, the relevance and usefulness of online learning communities have also been identified as an important factor in promoting student success. Similarly, a study by Ertl [43] found that students who perceived online learning communities as valuable were more likely to engage in collaborative learning and be successful in their courses. Finally, the importance of flexibility and adaptability in online learning communities has also been supported by previous research. A study by Shea, et al., [42] found that flexibility and adaptability were important factors in promoting student success in online learning environments. Additionally, a study by Garrison, et al., [36] found that online learning communities that were flexible and adaptable to the needs and preferences of students were more effective in promoting student success. Finally, Swan and Shih [44] found that students who perceived online learning communities as relevant and useful were more likely to participate and be successful in their courses.

## Conclusions

In conclusion, this multi-objective qualitative study has shed light on the impact and role of digital knowledge-sharing (DKS) communities in the development and innovation of online higher education. The study identified several benefits and challenges of using DKS communities in online education, as well as the ways in which these communities can contribute to higher education sustainability. The findings also revealed the role of DKS communities in facilitating collaboration and innovation, encouraging innovation, disseminating best practices, supporting lifelong learning, enhancing critical thinking, promoting student agency, and developing digital literacy. Moreover, the study highlighted the potential impact of DKS communities on student learning, including increased access to resources, peer support and collaboration, diverse perspectives, active learning, flexibility, and personalization. The study also identified important factors that promote student success in online learning communities, such as clear expectations and guidelines, supportive and collaborative learning environments, relevance and usefulness, and flexibility and adaptability. These findings have important implications for the development of online education and the use of DKS communities in higher education.

Despite the valuable insights provided by this multi-objective qualitative study, there are limitations that must be considered. First, the sample size of 20 informants may not be representative of the larger population, limiting the generalizability of the findings. Further studies with larger sample sizes are needed to confirm the results and provide more comprehensive insights into the impact and role of DKS communities in online higher education. Second, the use of a qualitative research approach may introduce researcher bias and limit the generalizability of the findings. Combining qualitative and quantitative methods could provide a more comprehensive understanding of the impact of DKS communities on online education. Finally, the study was conducted in a specific context, and the findings may not be applicable to other contexts. Future studies should consider contextual factors such as cultural differences and institutional policies to provide a more comprehensive understanding of the impact of DKS communities on online education. To address these limitations, future studies could employ quantitative research designs to provide more objective and generalizable results. Additionally, longitudinal studies could investigate the long-term impact of DKS communities on online education, providing insights into the sustainability of these communities. Comparative studies could also be conducted to compare the impact of DKS communities with other models of online education, identifying the strengths and weaknesses of DKS communities and providing insights into how to optimize their impact on online education. By addressing these limitations and exploring these suggestions, future studies can further advance our understanding of the impact and role of DKS communities in online higher education, informing best practices and contributing to the ongoing development and innovation of online education.

## Implications

First and foremost, it underscores the transformative potential of DKS communities in enhancing online education through the facilitation of collaboration, dissemination of best practices, and encouragement of innovation. These communities are poised to act as powerful catalysts for the continuous improvement of digital education. Secondly, the study places a spotlight on the paramount importance of addressing challenges such as quality control and bridging the digital divide to safeguard the long-term sustainability of DKS communities. Effective proactive strategies and purpose-built tools are imperative to unlock their full potential. Thirdly, DKS communities emerge as champions of lifelong learning, expanding horizons by broadening access to a wealth of diverse resources. Institutions stand to harness these communities to cater to a more extensive demographic of learners, thereby nurturing a culture of lifelong education. Fourthly, within the realm of online education, DKS communities assume a pivotal role in elevating critical thinking skills among students while fostering a sense of empowerment. The creation of environments that stimulate critical thought becomes indispensable in this context. Fifthly, the study underscores the necessity of establishing clear guidelines and expectations within DKS communities, serving as the linchpin for maximizing student engagement and success. The provision of structured and meticulously defined online learning environments emerges as the cornerstone of this endeavor.

### Supplementary Information

**Additional file 1: Appendix.** Interview checklist.

## Data Availability

The data would be available upon request from the corresponding author (email:Jiangtao20230628@163.com).
