# Supplementary material for: Research on the development and innovation of online education based on digital knowledge sharing community
Source: BMC Psychol. 2023 Sep 28;11:295. doi: 10.1186/s40359-023-01337-6 (PMC10540416; doi:10.1186/s40359-023-01337-6)
Supplement: Supplementary file 1 — Additional file 1: Appendix. Interview checklist. [file 40359_2023_1337_MOESM1_ESM.docx]

**Appendix**

Interview checklist

What motivated you to participate in the digital knowledge sharing community?

How do you think the digital knowledge sharing community has impacted your online education experience?

Could you describe some of the challenges you have faced while participating in the digital knowledge sharing community?

How do you think the digital knowledge sharing community could be improved to better support online education?

What do you think are the key benefits of digital knowledge sharing communities for online education?

In your opinion, what role do you think digital knowledge sharing communities play in promoting innovation in online education?

How do you think digital knowledge sharing communities can be effectively integrated into formal online education programs?

What are some of the limitations of digital knowledge sharing communities in the context of online education?

What do you think are the future prospects for digital knowledge sharing communities in online education, and how do you see them evolving over time?
